# Renal Replacement Therapy in Methylmalonic Aciduria-Related Metabolic Failure: Case Report and Literature Review

**DOI:** 10.3390/jcm13154304

**Published:** 2024-07-23

**Authors:** Giovanni Pintus, Nicola Vitturi, Gianni Carraro, Livia Lenzini, Giorgia Gugelmo, Ilaria Fasan, Alberto Madinelli, Alberto Burlina, Angelo Avogaro, Lorenzo Arcangelo Calò

**Affiliations:** 1 Hypertension Unit, Department of Medicine—DIMED, Padova University Hospital, University of Padova, 35128 Padua, Italy; giovanni.pintus@uniroma1.it (G.P.); livia.lenzini@unipd.it (L.L.); 2 Department of Clinical, Internal, Anesthesiological and Cardiovascular Sciences, Sapienza University of Rome, 00185 Rome, Italy; 3 Division of Metabolic Diseases, Department of Medicine, Padova University Hospital, University of Padova, 35128 Padua, Italy; nicola.vitturi@aopd.veneto.it (N.V.); giorgia.gugelmo@unipd.it (G.G.); alberto.maoinelli@aopd.veneto.it (A.M.); angelo.avogaro@unipd.it (A.A.); 4 Nephrology, Dialysis and Transplant Unit, Department of Medicine, Padova University Hospital, University of Padova, 35128 Padua, Italy; gianni.carraro_01@aopd.veneto.it; 5 Division of Clinical Nutrition, Department of Medicine—DIMED, Padova University Hospital, University of Padova, 35128 Padua, Italy; ilaria.fasan@unipd.it; 6 Division of Inherited Metabolic Diseases, Department of Women’s and Children’s Health, Padova University Hospital, University of Padova, 35128 Padua, Italy; alberto.burlina@unipd.it

**Keywords:** hemodialysis, metabolic crisis, methylmalonic aciduria, renal replacement therapy, rare disease, transplantation

## Abstract

Background: Methylmalonic Aciduria (MA) without homocystinuria (or isolated MA) is a group of rare inherited metabolic disorders which leads to the accumulation of methylmalonic acid (MMA), a toxic molecule that accumulates in blood, urine, and cerebrospinal fluid, causing acute and chronic complications including metabolic crises, acute kidney injury (AKI), and chronic kidney disease (CKD). Detailed Case Description: Herein, we report a case of a 39-year-old male with MA and stage IV CKD who experienced acute metabolic decompensation secondary to gastrointestinal infection. The patient underwent a single hemodialysis (HD) session to correct severe metabolic acidosis unresponsive to medical therapy and to rapidly remove MMA. The HD session resulted in prompt clinical improvement and shortening of hospitalization. Discussion: MMA accumulation in MA patients causes acute and life-threatening complications, such as metabolic decompensations, and long-term complications such as CKD, eventually leading to renal replacement therapy (RRT). Data reported in the literature show that, overall, all dialytic treatments (intermittent HD, continuous HD, peritoneal dialysis) are effective in MMA removal. HD, in particular, can be useful in the emergency setting to control metabolic crises, even with GFR > 15 mL/min. Kidney and/or liver transplantations are often needed in MA patients. While a solitary transplanted kidney can be rapidly affected by MMA exposure, with a decline in renal function even in the first year of follow-up, the combined liver–kidney transplantation showed better long-term results due to a combination of reduced MMA production along with increased urinary excretion. Conclusions: Early diagnosis, multidisciplinary management and preventive measures are pivotal in MA patients to avoid recurrent AKI episodes and, consequently, to slow down CKD progression.

## 1. Introduction

Methylmalonic Aciduria (MA) without homocystinuria (or isolated MA) is a group of rare inherited disorders resulting from mutations on the genes coding either for the mitochondrial enzyme methylmalonyl-CoA mutase (MCM) or for its cofactor 5’-deoxyadenosylcobalamin (AdoCbl). The overall prevalence is estimated to be 1/48,000–1/61,000 in North America and 1/26,000 in China [1]. Defects in MCM affect the conversion of methylmalonyl-CoA to succinyl-CoA, resulting in the accumulation of methylmalonic acid (MMA) [2]. MMA is a dicarboxylic acid with a molecular mass of 118.09 Da, detectable in blood, urine, and cerebrospinal fluid. Concurrent gastrointestinal disturbances, infectious disease, and catabolic state may trigger acute metabolic decompensation or crisis, and can be associated with high mortality rate, particularly in pediatric patients with severe MA forms, or in adults with compromised hepatic and/or renal function (chronic kidney disease, CKD) secondary to chronic MMA exposure [3]. In these cases, liver and/or kidney transplantation are frequently needed to manage metabolic decompensation and failure.

In recent decades, practices of prenatal [4] and neonatal [5] screening tests for MA have diffused worldwide, via the determination of organic acids and confirmed enzymatic or molecular genetic analyses [6]: prompt diagnosis, along with the early initiation of dietary and supplementary therapy, leads to overall reduced mortality from acute metabolic crises and increased life expectancy. However, long-term complications emerge due to chronic exposure to MMA, in particular at the levels of the central nervous system, bone marrow, and kidneys. In fact, these patients develop chronic tubulointerstitial nephropathy, which eventually lead to end-stage renal disease (ESRD) [3].

Herein, we present the case of an adult patient with a diagnosis of MA and stage IV CKD who required urgent dialysis for acute metabolic decompensation, and a review of the literature to evaluate MA clinical and therapeutic renal management.

## 2. Detailed Case Description

**Figure 1:**
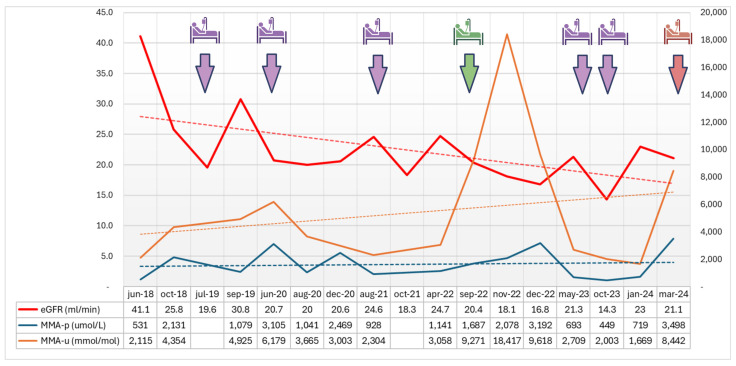
Trends over the last 6 years in renal function and methylmalonic acid levels. The raw data and trend for eGFR (mL/min, CKD-EPI formula) are reported in red; those for plasma and urinary methylmalonic acid (p-MMA, μmol/L; u-MMA, mmol/mol) are reported in blue and brown, respectively. Hospitalizations during the follow-up period are marked in purple for acute kidney injury on chronic kidney disease, green for worsening of peripheral edema, red for the hospitalization referred to in this case report. Data included from the referral of the patient to our Center for Metabolic Disorders (Azienda Ospedaliera di Padova, Padova, Italy) in 2018.

We report the case of G.N., a 39-year-old male diagnosed with MA (mut-subtype) at the age of 5 after a metabolic decompensation episode triggered by a protein-rich meal, affected also by CKD stage IV, with arterial hypertension, anemia, hyperuricemia, hyperparathyroidism (all secondary to CKD), hypothyroidism, and sensorimotor neuropathy in the lower limbs. The patient showed in recent years a gradual decline in renal function, and multiple hospitalizations (Figure 1): five were due to AKI on CKD, secondary to dehydration induced by profuse diarrhea and/or vomiting, and one was due to worsening peripheral edema. The patient followed a low-protein diet (2100 Kcal, proteins 0.67 g/kg: total protein 47 g) with amino acid supplementation (asadon xmtv 10 g × 2) and carnitine (carnitene 2 fl/day). Drug therapy was centered on vitamin B12 supplementation, allopurinol 150 mg/day, levothyroxine 75 mcg × 5 days, 50 mcg × 2 days, sodium bicarbonate 3 g × 3, sodium citrate/potassium citrate solution, cyanocobalamin 1 vial/week, atenolol 50 mg × 2, lacidipine 4 mg × 2, polystyrene sulfonate 1 dose × 3 days/week, cholecalciferol 10,000 IU 15 drops/week, epoetin alfa 4000 IU/15 days, furosemide 125 mg/day, and omeprazole 20 mg.

The patient was admitted to the emergency department, sent by his metabolic disease specialist, due to repeated and prolonged episodes of food vomiting in the last few days, along with low-grade fever, loss of appetite, fatigue, and a productive cough. At the first medical examination, vital parameters were within normal range, with no signs of edema. Other biometric data included body weight, 76.5 kg; height, 168 cm; and BMI, 27.1 kg/m^2^. Acute metabolic failure was suspected, likely triggered by a gastrointestinal infection.

Hematological and biochemical examinations are showed in Table 1. The patient started nutritional therapy with glucose solution and the correction of electrolyte imbalances and of metabolic acidosis, alongside antiemetic therapy and proton pump inhibitors. Blood examination showed neutrophilic leukocytosis with elevated CRP and empirical antibiotic therapy was started with ceftriaxone. Plasma ammonia was slightly increased, although comparable to pre-hospitalization levels; lactic acid was within the normal range; plasma carnitine was 71.9 μmol/L. Venous blood gas analysis revealed severe metabolic acidosis (pH 7.34, HCO3 −13.1 mEq/L, base excess −11.2 mEq/L, anion gap 33 mEq/L; Table 2), which was not responsive to bicarbonate therapy. Diuresis was preserved. MMA was tested on blood and urine; however, its dosage was not available in the acute setting. Later on, the report showed highly increased MMA levels (plasma MMA 3498 μmol/L, MMA 8442 μmol/L), several folds higher compared to the patient’s last control (plasma MMA 449 μmol/L, urinary MMA 2003 mmol/mol, respectively). Despite serum creatinine levels being comparable to the last assessment, a significant raise in urea was observed (creatinine 303 µmol/L, urea 29.5 mmol/L), indicating another episode of AKI on CKD.

Considering the clinical signs of acute metabolic failure, along with the severe metabolic acidosis, a few hours after hospitalization a hemodialysis session was indicated for the rapid removal of methylmalonic acid, to control the metabolic state, correct metabolic acidosis, and prevent further kidney damage. Viral markers were negative. Following the placement of a jugular Quinton catheter, a 3 h hemodialysis session was performed (CorDiax 5008, Fresenius ©, Bad Homburg, Germany) utilizing high-flux Classix FX100 membrane and A161 dialysis fluid (K^+^ 3 mmol/L, Ca^2+^ 1.5 mmol/L, Fresenius ©), with Qb 200 mL/min and UF/h 100 mL (no weight removal). Continuous glucose solution infusion was administered to maintain blood glucose > 120 mg/dL throughout the session.

The hemodialysis session occurred without complications, with a subsequent prompt remission of symptoms. During the following days, the patient reported clinical benefit from the therapy; there were no more vomiting episodes, and the low-grade fever and the neutrophil leukocytosis were no longer present after antibiotic therapy. Ammonia levels remained stable throughout hospitalization (32–35 μmol/L, peak 40 μmol/L).

After five days of hospitalization, the jugular Quinton catheter was removed and the patient was discharged. While he is currently not undergoing dialysis therapy, exams at follow-up visits confirmed the progression to stage V CKD (eGFR 14 mL/min), so he has been listed for kidney transplantation.

## 3. Discussion

MA represents a heterogeneous group of rare congenital disorders characterized by the accumulation of MMA and by a genotype–phenotype correlation [7]. The wide number of mutations recognized in the involved genes can, therefore, explain the heterogeneity observed in these patients in terms of clinical manifestations and responses to therapy; in particular, patients may present differences in their level of response to vitamin B12 supplementation, in the onset of the disease (from neonatal to late-onset), in the number and severity of the metabolic crisis, in the onset of CKD and development of ESRD, or in the timing for renal replacement therapy (RRT) and/or liver transplantation. Nevertheless, the accumulation of MMA in blood, urine, and cerebrospinal fluid leads to acute and chronic complications, even in early life, with a high mortality rate occurring in its severe forms: while prompt recognition of the disease led to overall reduced mortality in MA patients, Reischl-Hajiabadi et al. showed that neonatal screening may have a lesser impact on long-term outcomes in the most severe MA forms when compared both to cobalamin-responsive forms and to other inherited metabolic diseases [8].

### 3.1. CKD and ESRD in MA Patients

With the exception of milder forms (i.e., Cbl-A), over a long-term follow-up period, CKD develops almost inevitably in MA patients [3]. Hörster et al. [9] reported that their cohort of patients with mut0 (*n* = 42) and Cbl-B (*n* = 11) exhibited higher MMA excretion and an earlier onset of symptoms, a higher frequency of complications and deaths, and more frequent CKD (61% and 66%, respectively), which was predicted according to their urinary MMA levels, while better outcomes in mut- forms were observed by Liang et al., who reported that only 22 of 365 patients with such mutations had CKD [10].

An in-depth analysis of renal pathophysiology in MA was recently published by Morath et al. [3]. MMA may exert direct and indirect toxic effects [11], thus acting in the kidneys as a nephrotoxin, to which the epithelial cells are chronically exposed. Chronic tubular interstitial nephropathy sometimes develops with overt signs of tubular dysfunction [12]. Considering that AdoCbl is involved in the Krebs cycle, oxidative stress damage secondary to an increased level of reactive oxygen species (ROS) has been hypothesized in these patients [13], in particular at the level of renal tubular cells, and has been demonstrated both in vitro and in animal models [14,15,16]. Damage to the juxtaglomerular apparatus with consequent hyporeninemic hypoaldosteronism has also been hypothesized [17].

In the case reported here, chronic tubular interstitial nephritis developed in early life, with a progressive decline in renal function. Of note, various authors have suggested the use of cystatin C in MA clinical practice to monitor renal function. This could avoid the problems related to the loss of muscular mass during catabolic states, and relative decreases in serum creatinine levels [12,17]. Fortunately, our patient did not develop liver failure and did not require renal replacement therapy during almost 40 years of follow-up monitoring. Frequent episodes of gastrointestinal problems, such as episodes of vomiting and/or diarrhea, may easily result in AKI, as observed in our patient. Given the bidirectional relationship known between AKI and CKD, the decline in renal function may result accelerated. In our patient, episodes of AKI on CKD became more frequent in recent years and probably accelerated the decline in renal function, as observed in Figure 1.

### 3.2. Renal Replacement Therapy (RRT) in MA

During the last few decades, treatment options have led to improved survival, although a subsequent increase in severe long-term neurologic, renal, and hepatic complications has also been observed. Presence of ESRD and/or hyperammonemia are frequent indications for RRT in MA patients, in particular in Cbl-B and mut0 forms; on the other hand, RRT is rarely needed in Cbl-A. Cbl-C forms are often associated with AKI secondary to thrombotic microangiopathy, thus potentially requiring RRT for AKI; this has been reported in both infants and adults [18,19,20], and a case of eculizumab treatment has also been reported [21].

The first reports of RRT for MA were published almost fifty years ago. The first cases regarded mostly newborn patients requiring urgent dialysis. In 1979, an 8-month-old patient was treated with peritoneal dialysis [22], and in 1983, Moreno-Vega et al. described the case of a 2.5-year-old girl successfully treated with continuous peritoneal dialysis for 2 years [23]. However, various dialytic methods have been reported. The choice of dialytic procedure depends mostly on the age of the patient and on the protocols established in each center. It should be noted that, with MA being a rare disease, studies reporting on the efficacy of dialytic techniques are mostly retrospective [24] or within-patient analyses [25]. In 1996, Treacy et al. described a case of MA in a 7-year-old patient with glutathione deficiency and lactic acidosis who underwent hemodialysis to achieve clearance of propionic and methylmalonate metabolites; in this patient, serum and urinary MMA levels were almost completely cleared by HD [26]. Two years later, Van’t Hoff et al. described serum MMA clearance over a 22-week follow-up period in a 13-year-old patient who underwent chronic HD treatment for ESRD. The HD sessions cleared the patient’s MMA levels with efficacy, even when serum MMA levels later increased due to protein and calorie intake, which led to an increase in duration of the HD session [27]. More recently, Vernon et al. described in more detail the kinetics of methylmalonic acid during the HD session, in a 28-year-old patient with mut0 MA form and combined liver–kidney transplantation [28]. The patient was undergoing intermittent HD, and after a 3 h session (F18 filter, Fresenius ©) at Qb of 400 mL/min and Qb 600 mL/min, the MMA reduction ratio was 54.2%, with a plasma clearance of 103 mL/min and an estimated volume of distribution of 0.48 L/kg, similar to total body water; plasma MMA returned to baseline after 52 h. Chen et al., in 2009, reported on three male infants successfully treated with continuous artero-venous hemodiafiltration (HDF) for metabolic crisis (hemofilter FH22H, Gambro, Sweden; dialysate flow of 0.5 L/h with volumetric infusions pumps placed pre and post filter), and reported the detailed clearance of one patient: the MMA level of their 24 h urine sample was 582 mmol/day and their serum MMA was 136 mmol/L before dialysis, whereas these levels were 283 mmol/day and 56 mmol/L after, with a total of 2854 mmol MMA in the urine and dialysate per day [29]. Of note, Kido et al. [30] reported three pediatric MA patients that developed rhabdomyolysis after withdrawal from continuous hemodiafiltration, although it is not clear if there was a correlation with the technique of choice or with other factors. Tsai et al. compared intermittent HD (IHD) versus non-IHD in 15 patients with inborn errors of metabolism; while the authors reported the efficacy and the safety of IHD in rapidly removing different toxins, no significant differences were found in MMA removal between IHD and non-IHD [31]. In 2009, Etuwewe et al. reported a clearance of 950 μmol/day using continuous cycling PD [32]. While MMA clearance may be higher with HD when compared to PD, more data are needed to support this hypothesis. The advantages of PD should, however, always be considered: e.g., its suitability in pediatric patients and its continuous depurative action. In any case, all procedures of RRT (HD, HDF, PD, continuous HD/HDF) are clearly efficient in MMA removal. Plasma exchange, alone (used in a case report as an initial treatment [33]) or in combination with PD, has also been reported [34].

The dosage of MMA in blood and urine as a prognostic factor after RRT, or in the ambulatory setting during the follow-up period, may be limited by its physiological fluctuations in plasma (i.e., depending on the diet) and by the loss of renal function in urine. Thus, the efficacy of RRT can be better evaluated by achieving adequate control of the metabolic crisis and the related symptoms.

Dialysis has been previously used by a number of clinicians to achieve a good metabolic status prior to liver transplantation [35]. However, Kamei et al. did not observe any advantage with this practice in achieving a lower risk of metabolic decompensation [36].

In our patient, indication for RTT was strictly dependent on the metabolic crisis and the presence of concomitant factors (i.e., gastrointestinal infection) that would have further compromised the patient’s conditions, in particular regarding the incipient new episode of AKI on advanced CKD. In our patient, a more conservative approach could have been adopted in the absence of lactic acidosis and overt hyperammonemia, and in the presence of adequate diuresis under diuretic stimulation; however, in our patient, the severe metabolic acidosis was not responsive to treatment, while the patient was promptly responsive to only one HD session, which, removing MMA promptly, controlled the symptoms associated with the metabolic decompensation and overall led to a short period of hospitalization. Considering the costs from Diagnosis-Related Groups (DRGs), the medical fees in Regione Veneto, and the mean costs for hospitalization in Italy [37], the cost of one HD session and is lower than or equal to a day of hospitalization for patients with clinical conditions of such complexity and severity.

### 3.3. Transplantation in MA

The general paradigm of therapy in MA is represented by vitamin B12 supplementation, L-carnitine, and a diet low in isoleucine, valine, threonine, and methionine. Compliance with the therapy helps to reduce MMA levels in both the blood and urine, but even at a low quantity they still exert chronic damage in the kidney. The progressive reduction in GFR leads to decreased excretion of MMA, resulting in a vicious cycle of increased renal damage and consequent loss of renal function. Similarly, liver function may decrease over time, worsening acute metabolic decompensations. For these reasons, these patients are frequently considered for kidney and/or liver transplantation or combined transplantation [38]. As recently reported in a statement from the American College of Medical Genetics and Genomics (ACMG), severe early-onset disease with frequent episodes of metabolic decompensation is the most common indication for transplantation [39]; proposing a patient for liver and/or kidney transplantation represents a crucial moment that requires multidisciplinary expertise and a careful risk/benefit assessment. It should also be kept in mind that transplantation does not provide a definitive cure for a metabolic disorder and holds risks of complications related to the procedure and to intra-operatory metabolic decompensation [38,40]. Nevertheless, numerous authors have demonstrated improved outcomes after liver and/or kidney transplantation [28].

Usually, liver transplantation is required at a very early age: Dello Strologo and Cheng Lin both reported a median/mean age at transplantation of 1.8 years in their cohort of prevalently mut0 patients (23/24 and 10/11 mut0, respectively) [41,42], while Jiang et al. reported a mean age of 4.3 years (15 patients, all with mut-). Another series of studies, based on national or international cohorts of MA patients, showed how long-term favorable outcomes and better quality of life can be achieved [43], and although liver transplant may slow the decline in renal function in some forms [42], the benefit may be less clear in more severe cases [44].

The long-term efficacy of combined transplantation on renal function was observed by Dello Strogolo et al. in 2022, when the authors found in their cohort (with the majority of patients having mut0 and Cbl-B forms, which hold the worse prognoses) that after 1 year of follow-up monitoring, levels of MMA remained lower in patients with combined renal transplantation (*n* = 33) or liver transplantation (*n* = 24) compared to kidney transplantation alone (*n* = 26) [41]; these results align with those previously published by Brassier et al. on a less numerous French cohort [45]. Moreover, patients that underwent solitary kidney transplantation showed lower eGFR after one year of follow-up monitoring when compared to those that underwent liver transplantation or combined liver–kidney transplantation and to patients who received kidney transplantation for another primary kidney disease (CERTAIN registry) [41,46]. The possible conclusion is that higher enzymatic activity provided by liver transplantation holds long-term benefits, compared to the improved excretion of MMA in solitary-kidney-transplantation patients, which, however, will remain eventually affected by the persistence of high MMA levels. Chakrapani et al. [38] reported that almost half of the patients that underwent kidney transplantation alone had ESRD as an indication, while, as proposed by the same authors, it is advisable to favor early liver transplantation to prevent long-term complications and to favor combined kidney–liver transplantation in the presence of overt and progressing kidney damage.

## 4. Conclusions

In conclusion, dialysis is efficient for MMA removal in MA patients, and in some patients can be prescribed in the emergency setting to control metabolic crises, even with GFR > 15 mL/min. The treatment of choice should depend on the patient’s clinical status and characteristics (such as their age); standard HD might represent the optimal compromise in patients, such as the one we have reported. Long-term outcomes are improved by liver or combined kidney–liver transplantation, while solitary kidney transplantation is rapidly affected by MMA exposure. During the strict follow-up monitoring of MA patients with CKD, the prevention of AKI episodes secondary to dehydration is essential in order to limit the decline in kidney function.

## Figures and Tables

**Table 1:** Comparison of blood and urine tests at admission and discharge.

Blood and Urine Tests	Admission	Discharge
White cells (10^9^/L)	14.16	6.35
Neutrophils (10^9^/L)	12.35	7.04
Red cells (10^12^/L)	3.67	3.42
Hemoglobin (g/L)	105	97
Hematocrit (L/L)	0.329	0.304
MCV (fL)	89.6	88.9
MCH (pg)	28.6	28.4
MCHC (g/L)	319	319
Platelets (10^9^/L)	233	242
P-carnitine (µmol/L)	71.9	
Creatinine (µmol/L)	303	424
Urea (mmol/L)	29.5	20.7
Sodium (mmol/L)	142	139
Potassium (mmol/L)	3.8	4.7
Chloride (mmol/L)	95	96
Calcium (mmol/L)	2.74	2.45
Phosphorus (mmol/L)	1.19	1.42
Magnesium (mmol/L)	0.76	0.86
Lactate dehydrogenase (U/L)	241	255
Lactic acid (mmol/L)	2	2
Ammonia (µmol/L)	32	35
C-reactive protein (mg/L)	14.84	
Procalcitonin (ug/L)	0.62	0.49
Glucose (mmol/L)	9.9	4.2
Intact PTH (ng/L) (n.v. 6.5–36.8)	123	
Urinary pH	6	7
Urinary proteins (g/L)	1	0.3
Urinary albumin/creatinine (mg/g)	>300	>300

**Table 2:** Comparison of venous blood gas analysis at admission and discharge.

Venous Blood Gas Analysis	Admission	Day 2	Day 4	Discharge
pH	7.34	7.39	7.39	7.43
HCO_3_^−^ (mmol/L)	13.1	23.2	25.5	26.9
Base excess (mmol/L)	−11.2	−1.5	0.4	2.4
Anion gap (mmol/L)	33	14	18	18

## Data Availability

Data are contained within the article.
